# Molecular Mechanisms and Signaling Pathways Underlying the Therapeutic Potential of Thymoquinone Against Colorectal Cancer

**DOI:** 10.3390/molecules29245907

**Published:** 2024-12-14

**Authors:** Amin F. Majdalawieh, Saud Al-Samaraie, Tala M. Terro

**Affiliations:** 1 Department of Biology, Chemistry, and Environmental Sciences, College of Arts and Sciences, American University of Sharjah, Sharjah P.O. Box 26666, United Arab Emirates; g00086819@alumni.aus.edu; 2 School of Medicine, University College Dublin, Belfield, D04 V1W8 Dublin, Ireland; saud.alsamaraie@ucdconnect.ie

**Keywords:** thymoquinone, colorectal cancer, anti-cancer, antioxidant, anti-proliferative

## Abstract

Thymoquinone (TQ), a bioactive compound derived from *Nigella sativa*, has garnered significant attention for its potential as a natural anti-cancer agent, particularly in the context of colorectal cancer. This review provides a detailed synthesis of the current literature on the anti-cancer properties of TQ in colorectal cancer cells, exploring both in vitro and in vivo studies to elucidate its mechanisms of action. TQ effectively induces apoptosis, inhibits cell proliferation, and reduces metastasis in colorectal cancer cells by modulating key molecular pathways such as PI3K/AKT/mTOR, NF-κB, STAT3, and MAPK. It causes mitochondrial dysfunction and activates caspases, contributing to its pro-apoptotic effects. TQ also regulates EMT and targets cancer stem cells, reducing the likelihood of metastasis. Moreover, its antioxidant properties contribute to its protective role against cancer progression. While preclinical studies provide strong evidence of TQ’s efficacy, further clinical studies are essential to establish its therapeutic potential in humans. This review underscores TQ’s promising role as a natural agent with the potential to significantly improve colorectal cancer treatment outcomes.

## 1. Introduction

Colorectal cancer (CRC) is one of the most prevalent and lethal forms of cancer worldwide, ranking as the third most common cancer and the second leading cause of cancer-related deaths [1,2]. Despite advances in early detection, surgical interventions, and chemotherapeutic regimens, the prognosis for CRC patients, particularly those diagnosed at advanced stages, remains poor [3]. Tumor metastasis, recurrence, and resistance to standard therapies such as 5-fluorouracil (5FU) and irinotecan present significant challenges, emphasizing the urgent need for novel therapeutic strategies that can improve patient outcomes [4].

Natural compounds have garnered increasing attention in cancer therapy due to their potent bioactive properties and relatively low toxicity compared to traditional chemotherapy [5]. Among these, thymoquinone (TQ), the active ingredient of *Nigella sativa* (black seed), has emerged as a promising candidate [6,7]. It has a molecular weight of 164.204 g/mol and bears the chemical formula C_10_H_12_O_2_. Traditionally, *Nigella sativa* has been utilized in various forms to treat a wide range of ailments, including asthma, hypertension, diabetes, inflammation, cough, bronchitis, headaches, eczema, fever, dizziness, and influenza [8]. Experimental studies have shown that extracts from *N. sativa* and its primary volatile oil component, TQ, exhibit significant antioxidant, anti-inflammatory, and hepatoprotective properties [9,10]. Its ability to target cancer cells selectively, sparing normal cells, makes it an attractive option for cancer treatment [11,12]. The therapeutic potential of TQ in CRC, specifically, has been a focal point of recent studies, which have revealed its ability to modulate critical molecular pathways involved in cancer progression [13].

This review aims to provide a comprehensive overview of the anti-cancer effects of thymoquinone on colorectal cancer, focusing on its anti-proliferative [14], pro-apoptotic [15], anti-metastatic [16], antioxidant effects [17], and the underlying signaling pathways involved [18]. It explores how TQ influences key signaling pathways such as the phosphoinositide 3 kinase (PI3K)/protein kinase B (Akt)/mammalian target of rapamycin (mTOR), nuclear factor kappa B (NF-κB), signal transducer and activator of transcription 3 (STAT3), and mitogen-activated protein kinase (MAPK) pathways, which are crucial in cancer cell survival, proliferation, and metastasis. Moreover, the review will highlight TQ’s role in modulating glucose metabolism, inhibiting the Warburg effect, and promoting apoptosis and cell cycle arrest in CRC cells. In addition to its apoptotic effects, we delve into its anti-metastatic actions, including the inhibition of epithelial-to-mesenchymal transition (EMT) and reduction of cancer stem cell (CSC) sphere formation. The ability of TQ to induce oxidative stress, modulate antioxidant defenses, and affect tumor microenvironment components will also be discussed.

Furthermore, this review will examine the synergistic effects of TQ when combined with traditional therapies such as ionizing radiation (IR) and chemotherapeutic agents like cisplatin, revealing its potential to overcome chemoresistance and enhance treatment efficacy. The promising results from in vitro, in vivo, and ex vivo studies underscore TQ’s potential as a multi-targeted therapeutic agent in colorectal cancer treatment.

Given the evidence supporting TQ’s anti-cancer properties, understanding the specific mechanisms by which TQ exerts its effects could pave the way for the development of novel therapeutic strategies that improve clinical outcomes for CRC patients. This review, therefore, aims to summarize the current state of research on TQ’s anti-cancer mechanisms in colorectal cancer, offering insights into its potential for integration into clinical practice as an adjunctive or alternative treatment modality.

## 2. Search Methodology

The literature search was conducted using a variety of online databases, such as PubMed, Elsevier (Science Direct), and Google Scholar. To ensure the identification of relevant studies, the word “thymoquinone” was used along with keywords including “colorectal cancer”, “anti-cancer”, and “antioxidant”. Articles were screened and selected, with no restriction on publication dates, the experimental subjects/models used, mode and duration of thymoquinone administration, or other experimental details.

## 3. Anti-Proliferative and Pro-Apoptotic Effects of TQ

TQ has shown remarkable anti-proliferative and pro-apoptotic effects across a variety of colorectal cancer cell lines. In HCT116 cells, TQ (60 μM) reduced cell viability in a dose-dependent manner, with an IC_50_ of 68 μM [14]. In vitro studies by Tabet-Helal [19] further demonstrated that TQ (60 μM) led to a substantial increase in cell mortality—78.8% ± 8.48 compared to 28.2% ± 6.38 in the control group. This effect correlated with a decrease in cell proliferation, as evidenced by reduced bromodeoxyuridine (BrdU) absorbance. Morphological changes such as elevated p53 expression and myeloid cell leukemia sequence-1 (Mcl-1) downregulation were also observed [19]. In addition, treatment with varying concentrations of TQ (12.5, 25, 50, and 100 μg/mL) resulted in a reduction of over 40% in cell viability in HCT116 cells and 35% in HT-29 cells at 50 μg/mL, without harming non-malignant colonic cell [20,21]. Supporting these findings, Fröhlich et al. [22] showed that TQ (1, 2.5, and 5 μM) increased poly (ADP-ribose) polymerase (PARP) cleavage in HCT116 cells after 48 h of treatment, while TQ (40 and 60 μM) induced apoptosis through increased TUNEL positivity and upregulation of key apoptosis-related proteins, including p53, p21, and γ-H2AX. Furthermore, in NOD-SCID and NOG mice, TQ (20 mg/kg) effectively reduced tumor volume in both 5FU-sensitive and resistant spheres, with no significant toxicity [16]. Ballout and colleagues [16] also found that TQ (1, 3 and 5 µM) decreased the expression of key stem cell markers (CD44 and EpCAM) and proliferation marker Ki67, indicating reduced self-renewal capacity.

Interestingly, TQ (20, 40, and 60 μM) significantly downregulated checkpoint kinase 1 (CHEK1), which coordinates the DNA damage response and cell cycle checkpoint response, in p53^+/+^ HCT116 cells but upregulated it in p53^−/−^ cells, suggesting that p53 represses CHEK1 expression. CHEK1 repression contributed to TQ-induced apoptosis, as restoring p53 in p53^−/−^ cells led to lower CHEK1 levels and increased caspase-3 activity. This downregulation occurred through the direct binding of p53 to the CHEK1 promoter [23]. In xenograft models with male NMRI mice, tumors expressing functional p53 (p53^+/+^) had lower CHEK1 expression and higher rates of apoptosis in response to TQ (20 mg/kg) compared to p53^−/−^ tumors. Clinical samples corroborated these findings, showing that higher CHEK1 expression was linked to advanced tumor stages, distal localization, and poor prognosis. These results highlight the role of TQ in inducing p53-dependent apoptosis by repressing CHEK1, while elevated CHEK1 expression is associated with more aggressive colorectal cancers and worse patient outcomes [23].

**Figure 1:**
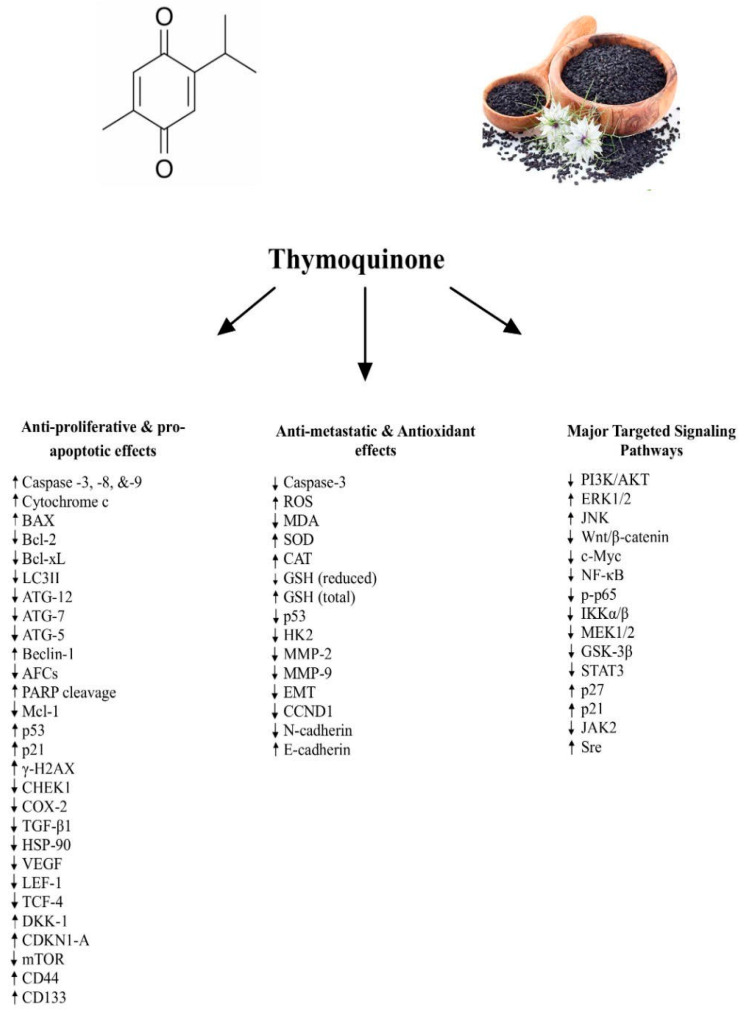
A schematic representation of the major molecular and cellular mechanisms and targeted signaling pathways underlying the anti-metastatic, pro-apoptotic/anti-proliferative, and antioxidant activities of TQ.

Further reinforcing these insights, Tokay and colleagues [24] demonstrated that TQ (9, 18, 39, 75, and 150 µM) decreased HT29 cell viability in a dose- and time-dependent manner over 24 and 48 h. At the highest dose (150 µM), TQ significantly reduced the invasive abilities of HT-29 cells by approximately eight-fold compared to untreated cells within 6 h. Additionally, TQ (150 µM) significantly downregulated autophagy-related proteins such as ATG-12, ATG-7, and LC3-II, indicating its impact on autophagy processes. Notably, TQ treatment at 150 µM for 48 h also led to a 15-fold increase in the pro-apoptotic gene Bcl-2-associated protein x (Bax) while decreasing the expression of anti-apoptotic proteins B cell lymphoma/leukemia type 2 (Bcl-2) and B-cell lymphoma-extra-large (Bcl-xl) [24] (Figure 1).

In HCT116 cells, treatment with TQ (100 μM) led to a three-fold increase in p53 protein levels compared to untreated controls, alongside an upregulation of p53 messenger ribonucleic acid (mRNA) and a decrease in Bcl-2 mRNA expression [25]. Moreover, TQ (100 μM) induced cell cycle arrest at the growth phase-1 (G1)/S phase, accompanied by an increased number of cells in the pre-G1 phase, indicating apoptosis, which was further confirmed by a dose-dependent rise in p53 expression. In another study, the chemoprotective and anti-carcinogenic properties of TQ were evaluated in familial adenomatous polyposis (FAP) mice. Over a 12-week period, TQ (375 mg/kg) significantly reduced the number of large polyps in the small intestine, although it had limited effects on tumor multiplicity in Apc^Min^ mice. TQ (375 mg/kg) selectively induced apoptosis in neoplastic cells within polyps, significantly increasing the number of apoptotic cells compared to untreated mice. However, cell proliferation, measured by Ki-67 staining, was not altered in either polyps or normal tissue [12].

Interestingly, Lang et al. [12] showed that the effectiveness of TQ was found to be influenced by the mutational status of adenomatous polyposis coli (APC) and p53, with varying sensitivities across cell lines. Colon cancer cells harboring mutations in both APC and p53 (DLD1 and HT29) were most resistant to TQ, exhibiting high IC_50_ values (196 μM and 160 μM, respectively). In contrast, LoVo cells, which carry wild-type *p53* but a mutated *APC gene*, were most sensitive to TQ (IC_50_: 36 μM). Cells with both wild-type *p53* and *APC*, such as HCT116, RKO, and HCEC-1CT, displayed moderate sensitivity, with IC_50_ values ranging from 79 to 118 μM. In a related study by, TQ (75 μM) significantly induced apoptosis, reflected by a nine-fold increase in cells at the sub-G1 phase, while enhancing the expression of p21, a cyclin-dependent kinase inhibitor that impedes cell cycle progression, in HT29, SW480, and SW620 cells [26].

Mohamed et al. [27] demonstrated that Wistar rats injected with azoxymethane (AOM) developed numerous colorectal tumors and abnormalities, however, treatment with TQ (35 mg/kg/day) significantly mitigated these effects. Histopathological analysis confirmed that TQ treatment markedly reduced AOM-induced large aberrant crypt foci (ACF) and tubular adenomas. Mohamed et al. [28] also demonstrated that TQ (35 mg/kg/day) significantly reduced total tumor counts to 17.6 ± 2.2 units compared to 29.16 ± 2.92 units in the AOM control group. Additionally, TQ (35 mg/kg/day) reduced large ACFs and downregulated key genes involved in colon carcinogenesis, including Wnt, β-catenin, NF-κB, and cyclooxygenase-2 (COX-2), while upregulating tumor suppressor genes such as dickkopf-1 (DKK-1) and cyclin dependent kinase inhibitor 1A (CDKN1-A). Protein analysis via ELISA revealed lower levels of transforming growth factor β1 (TGF-β1), COX-2, heat shock protein 90 (HSP-90), and vascular endothelial growth factor (VEGF) in TQ-treated groups, while immunohistochemical analysis confirmed reduced smad4 expression and increased inducible nitric oxide synthase (iNOS) in AOM-treated tissues, effects that were counteracted by TQ treatment [28].

In LoVo cells, Hsu and colleagues [13] reported that TQ (5, 10, and 20 μM) suppressed the expression of proteins critical for cancer progression, including COX-2, PI3K, AKT, and glycogen synthase kinase-3 beta (GSK-3β), with the strongest reduction observed at 20 μM. Further analysis showed that TQ inhibited the translocation of β-catenin into the nucleus and decreased levels of β-catenin and its co-interacting partners, lymphoid enhancer-binding factor 1 (LEF-1) and transcription factor 4 (TCF-4), which are key players in promoting tumorigenesis. In vivo studies corroborated these findings, revealing that treatment with prostaglandin E2 (PGE2) and TQ (20 μM) significantly reduced β-catenin and COX-2 levels [13].

Additionally, Chen and colleagues [29] observed that TQ (2, 4, 6, and 8 μM) induced a dose-dependent increase in total Lovo cell death, with early apoptosis markers such as cytochrome c, caspase 9, and caspase 3 rising at low doses (2 μM). However, at higher doses (8 μM), apoptosis diminished as autophagy markers increased. At 8 μM, TQ induced mitochondrial outer membrane permeabilization (MOMP) and elevated autophagy-related proteins, including ATG-5, ATG-7, Beclin-1, LC3, lysosome-associated membrane glycoprotein 2 (LAMP2), and sequestosome-1 (SQSTM1)/p62, confirming the presence of autophagic cell death (ACD) in Lovo cells. Activation of jun N-terminal kinase (JNK) and p38 kinases mediated this effect, as inhibitors SP600125 and SB203580 reversed TQ’s impact, restoring apoptosis while reducing autophagy markers. Furthermore, autophagy inhibitors (3-MA and LY294002) suppressed autophagy markers and restored caspase 3 activation, indicating that TQ initially triggers apoptosis before switching to ACD during autophagosome formation [29].

In an in vitro study by Kundu et al. [30], annexin V staining revealed that TQ (10, 25, and 50 μM) induced apoptosis in a concentration-dependent manner in HCT-116 cells, as indicated by decreased levels of Bcl-2 and Bcl-xl and increased levels of Bax. TQ treatment also led to the cleavage of caspase-9, caspase-7, and caspase-3, as well as PARP, with a corresponding increase in caspase-3 activity (Figure 1). El-Najjar et al. [31] further explored the effects of TQ in HT-29, HCT-116, DLD-1, LoVo, and Caco-2 cells, reporting dose- and time-dependent cytotoxicity, with TQ (20, 40, 60, and 100 μM) showing higher toxicity in cancer cells than in normal intestinal cells. DLD-1 cells, in particular, exhibited a marked increase in apoptosis via ROS generation following TQ (40 μM) treatment, accompanied by elevated caspase-3 and caspase-7 activity and accumulation in the pre-G1 phase of the cell cycle. In HT-29 cells, resistance to TQ (40 μM) was partially attributed to high levels of DT-diaphorase, an enzyme that diminished TQ's effectiveness. Inhibition of DT-diaphorase with dicumarol sensitized HT-29 cells to TQ, enhancing its apoptotic effects. However, in the in vivo study by Jrah-Harzallah et al. [32], in male Wistar rats, 5 mg/kg of TQ exerted an antioxidant effect during 1,2-dimethylhydrazine (DMH)- induced carcinogenesis. TQ pre-treatment completely restored DMH-induced lipid peroxidation and antioxidant levels, and instead of increasing, it attenuated ROS generation. This discrepancy, when compared to the El-Najjar et al. [31] study, could be attributed to the dual nature of TQ as being both a prooxidant (pro-apoptotic) and an antioxidant (anti-proliferative) [33]. The vast majority of studies show that TQ increases ROS generation, contributing to its anticancer properties. The effects of TQ on ROS levels are highly dependent on factors such as dosage and the cellular/experimental context (in vitro vs. in vivo), which can include differences in metabolic compounds or redox behaviors within each environment [34]. 

Zhang et al. [35] investigated the potential of TQ to enhance chemosensitivity in colon cancer cells (HCT116 and COLO205). TQ (20 μM in HCT116 and 40 μM in COLO205) demonstrated dose-dependent cytotoxic effects, significantly reducing cell viability. Combining TQ (20 or 40 µM) with cisplatin (CisPt) further amplified cell death in both cell lines compared to cisplatin alone, suggesting that TQ could potentiate the chemosensitivity of colon cancer cells.

Al Bitar et al. [36] conducted both in vitro and ex vivo studies, showing that TQ, either alone or in combination with IR, reduced colorectal cancer cell proliferation in a time- and dose-dependent manner. TQ (40 or 60 μM) significantly suppressed proliferation in HCT116 cells after 48 h of treatment, while in DLD1 cells, the combination of TQ (60 μM) and IR further reduced proliferation. This effect was also observed in HT29 cells, albeit at a higher TQ concentration (120 μM). The combined TQ and IR treatment led to a significant reduction in cell viability and colony formation compared to either treatment alone. Specifically, TQ (10–60 μM) combined with IR markedly inhibited long-term survival in HCT116 and HT29 cells. The combination also induced G2/M cell cycle arrest by activating the DNA damage response via γH2AX and the kinases ataxia-telangiectasia mutated (ATM) and ataxia telangiectasia and Rad3-related (ATR). Additionally, the combined treatment suppressed survival and growth signaling proteins mTOR, mitogen-activated protein kinase kinase (MEK), and NF-κB and stem cell markers (CD133, β-catenin, CD44), indicating enhanced DNA damage and inhibition of stem cell-like properties. Furthermore, the combination slightly increased p53 expression and upregulated p21, reinforcing its apoptotic effects [36].

In vivo, Gali-Muhtasib et al. [15] reported that TQ (5 mg/kg) significantly reduced ACF by 86% and decreased tumor development in female BALB/c mice compared to 1,2-dimethylhydrazine (DMH)-treated controls. Histological analysis revealed that TQ-treated mice developed well-differentiated adenocarcinomas with increased apoptosis, as evidenced by caspase-3 and TUNEL staining.

Kortüm et al. [37] studied HCT116 cells and found that TQ (2.5 μM) improved replication fidelity and reduced cell growth. TQ (37.5 and 375 mg/kg) also reduced small intestinal tumor incidence from 94% to 56% in Msh2^loxP/loxP^ Villin-Cre mice and decreased tumor multiplicity and mutation rates in tumor tissues, showing a significant reduction in microsatellite mutations. Importantly, these effects were independent of apoptosis or antiproliferative actions, suggesting that TQ may serve as a chemopreventive agent in small intestinal and caecal tumorigenesis.

## 4. Anti-Metastatic and Antioxidant Effects of TQ

Several studies have explored the anti-metastatic potential of TQ. Hsu et al. [13] found that TQ (5, 10, and 20 μM) significantly reduced LoVo cell migration in a dose-dependent manner, highlighting its potential to inhibit metastasis. Moreover, Gali-Muhtasib et al. [15] showed that TQ (20, 40, 60 μM) reduced cell invasion potential of C26 cells. Similarly, Ballout et al. [16] demonstrated that TQ (40 and 60 μM) markedly inhibited migration and invasion in colorectal cancer cells, which was associated with the upregulation of the epithelial marker epithelial (E)-cadherin and the downregulation of mesenchymal markers. Moreover, TQ (21.71 µM in HCT116 and 20.53 µM in SW480) exhibited anti-metastatic effects by disrupting glycolytic metabolism through modulation of the PI3K-AKT pathway, impacting glucose metabolism [18]. Ballout et al. [16] also showed that TQ (1, 3, and 5 μM) reduced the sphere formation ability of CSCs derived from both 5FU-sensitive and resistant cells, further demonstrating its anti-metastatic properties.

In a 3D culture model, Al Bitar et al. [36] reported that TQ (3 µM), combined with IR, significantly reduced the sphere-forming ability of CRC cell lines, with stronger effects observed across successive generations. Patient-derived organoids (PDOs) from CRC patients exhibited a similar response, where the combination of TQ and IR reduced both organoid count and size more effectively than individual treatments. Additionally, Idris et al. [26] found that TQ (75 μM) led to the lowest expression levels of the cyclin D1 (CCND1) gene, which is associated with reduced metastasis in colorectal cancer cells (HT29, SW480, and SW620).

Chen et al. [38] found that TQ (2, 4, 6, and 8 μM) suppressed EMT and metastasis by reducing levels of key EMT markers, including Snail, Twist, matrix metalloproteinase-2 (MMP-2), and matrix metalloproteinase-9 (MMP-9). In migration and invasion assays, TQ (2, 4, 6, and 8 μM) significantly inhibited the migratory and invasive abilities of CPT-11-resistant cells, reducing migration by up to 79.26% and invasion by 92.83% compared to controls. Karim et al. [18] further observed that TQ (21.71 µM in HCT116 and 20.53 µM in SW480) exhibited anti-metastatic effects by upregulating E-cadherin and downregulating neural (N)-cadherin (Figure 1). Moreover, the genetic ablation of hexokinase 2 (HK2), a key regulator during glucose metabolism linked to malignant growth in many types of cancers, also reduced metastasis, suggesting that TQ’s anti-metastatic properties may be linked to its ability to modulate glucose metabolism.

TQ has also demonstrated significant antioxidant properties. In vitro, Mahdy et al. [17] found that TQ (389.60 µg/mL) decreased the reduced form of glutathione (GSH), dropping from 0.4442 ± 0.15 mM in control cells to 0.0222 ± 0.02 mM after treatment in Caco2 cells. An in vivo study by Jrah-Harzallah et al. [32] evaluated TQ’s effects on DMH-induced oxidative stress during colon carcinogenesis in male Wistar rats; TQ (5 mg/kg) significantly inhibited tumor development, reduced oxidative stress markers [ROS, malondialdehyde (MDA), and conjugated dienes], and restored antioxidant enzyme levels [glutathione peroxidase (GPx), catalase (CAT), superoxide dismutase (SOD), and reduced GSH].

Mahdy et al. [17] and Jrah-Harzallah et al. [32] examined the reduced form of GSH, which is the active antioxidant that provides protection against H_2_O_2_ for colorectal cancer (CRC) cells. To further elucidate TQ’s impact on cellular redox state, Idris et al. [26] examined the total GSH levels (which includes reduced GSH and oxidized GSH or glutathione disulfide or GSSG) to provide further insight into how levels of reduced or total GSH impact the antioxidant capacities of CRC and how TQ may mitigate such effects. Idris et al. [26] found that TQ (75 μM) reduced hydrogen peroxide (H_2_O_2_) levels while increasing total GSH, thus enhancing oxidative stress modulation in HT29, SW480, and SW620 cells (Figure 1). This approach is important, as it provides evidence of GSH’s potential pathogenic role in cancer progression and provides insight into TQ’s roles in both modulating oxidative stress and enhancing oxidative defenses against CRC cells [39].

## 5. Signaling Pathways Underlying the Anti-Cancer Effects of TQ

TQ has been shown to influence several key signaling pathways in colorectal cancer. Its impact on the PI3K/AKT pathway is well-documented, particularly in disrupting cancer cell metabolism. In a study by Karim et al. [18], TQ (21.71 µM in HCT116 and 20.53 µM in SW480) inhibited glucose fermentation, adenosine triphosphate (ATP) production, and nicotinamide adenine dinucleotide phosphate (NADPH) generation, effectively disrupting the Warburg effect, a phenomenon where cancer cells shift from oxidative phosphorylation to glycolysis; this modulation of glucose metabolism involved the suppression of HK2. Similarly, Idris et al. [26] found that TQ (75 μM) reduced the tumor suppressor gene phosphatase and tensin homolog deleted on chromosome 10 (PTEN) expression and inhibited the PI3K/AKT/mTOR pathway in HT29, SW480, and SW620 cells, thereby reducing tumor growth (Figure 1).

TQ also modulates the NF-κB and STAT3 pathways, both of which are implicated in cancer progression and survival. El-Far et al. [40] demonstrated that TQ (30 μM) increased levels of Bax and decreased Bcl-2 in HCT116 cells while inhibiting NF-κB and STAT3. These effects were further corroborated in vivo, where TQ-treated female NCr nude mice exhibited significant tumor size reduction with no detectable viable cancer cells. In another study, Zhang et al. [35] found that TQ (60 μM) inhibited NF-κB activation by reducing the phosphorylation of its p65 subunit in COLO205 cells, leading to the downregulation of survival-related proteins such as VEGF, cellular (c)-Myc, and Bcl-2. Furthermore, the combination of TQ (40 µM) and cisplatin with an NF-κB inhibitor (PDTC) led to enhanced cancer cell growth inhibition, underscoring the chemosensitizing effect of TQ via NF-κB inhibition.

Chen et al. [38] supported these findings by showing that TQ (2, 4, 6, and 8 μM) reduced the protein levels and phosphorylation of NF-κB and inhibitor of nuclear factor kappa B subunit alpha/beta (IKKα/β) in a dose-dependent manner. The highest concentration (8 μM) had the most profound tumoricidal effects, and immunofluorescence assays further confirmed NF-κB inhibition. Kundu et al. [30] revealed that TQ (10, 25, and 50 μM) inhibited the activation of STAT3 by reducing its phosphorylation at tyrosine-705, decreasing its nuclear localization, and lowering STAT3 reporter gene activity. This led to a reduction in the expression of STAT3 target genes (survivin, c-Myc, cyclin D1, and cyclin D2) while upregulating cell cycle regulators such as p27 and p21. Moreover, TQ diminished the phosphorylation of upstream kinases, including epidermal growth factor receptor (EGFR), janus kinase 2 (JAK2), and Src. Inhibition of these kinases by specific inhibitors (AG490, PP2, and gefitinib) further validated their role in STAT3 activation.

TQ’s effect on the MAPK pathway is another key area of focus. El-Najjar et al. [31] reported that TQ (20, 40, and 60 μM) activated the extracellular signal-regulated kinase (ERK) and JNK pathways in DLD-1 cells through ROS production. When these pathways were inhibited using PD98059 and SP600125, TQ’s pro-apoptotic effects were enhanced, suggesting that MAPK inhibition sensitizes cancer cells to TQ-induced apoptosis. The combination of TQ and MAPK inhibitors led to increased apoptosis and higher caspase-3 activity, further highlighting the significance of these pathways in TQ-mediated cell death.

Additionally, TQ has been found to modulate the β-catenin pathway. In a study involving FAP, Lang et al. [12] demonstrated that TQ reduced nuclear c-Myc protein expression in polyps, indicating downregulation of the β-catenin pathway. This was further confirmed in RKO cells, where TQ (30 μM) reduced nuclear β-catenin while increasing its cytoplasmic and membranous levels. Furthermore, TQ inhibited the phosphorylation of GSK-3β through suppression of the MEK1/2 pathway, leading to reduced c-Myc protein levels without affecting its mRNA expression (Figure 1).

Figure 1 provides a schematic representation of the known molecular and cellular mechanisms as well as the major targeted signaling pathways underlying the reported anti-cancer effects of TQ.

Table 1 presents a detailed summary of the reported anti-cancer activities of TQ, the dosages, the dosage regimens, the experimental models, as well as whether the reported effects are dose-dependent and/or time-dependent.

## 6. Conclusions

The findings of this review emphasize the promising potential of thymoquinone as a natural anti-cancer agent, particularly in the context of colorectal cancer. Through a comprehensive evaluation of in vitro and in vivo studies, TQ has demonstrated significant cytotoxicity against colorectal cancer cells while exhibiting minimal toxicity toward normal cells. The compound’s ability to target and disrupt key molecular pathways involved in cancer progression, such as the PI3K/AKT/mTOR, NF-κB, STAT3, and MAPK signaling pathways, highlights its efficacy in suppressing cancer cell proliferation, promoting apoptosis, and inhibiting metastasis. While preclinical studies have provided substantial evidence for TQ’s anti-cancer properties, further clinical studies are necessary to validate these findings in humans. Understanding the pharmacokinetics, optimal dosing regimens, and potential side effects will be crucial for translating these promising results into clinical practice. With ongoing research, TQ holds significant promise for contributing to the advancement of colorectal cancer treatment and improving patient outcomes.

## Figures and Tables

**Table 1:** A detailed summary of the main findings related to the anti-cancer properties of TQ, the dosages, the dosage regimens, the experimental models, as well as whether the reported effects are dose-dependent and/or time-dependent.

Main Effects	Experimental Model	Dosage	Administration Mode and Duration	Dose-Dependent and/or Time-Dependent	References
TQ reduced cellular proliferation TQ inhibited cell viability and colony formation with ionizing radiation (IR) TQ reduced mTOR, MEK, and NF-κB and stem cell markers CD133, β-catenin, and CD44 TQ induced G2/M cell cycle arrest TQ increased p53 expression and upregulated p21 expression TQ and IR reduced the sphere-forming ability of CRC cells	HCT116, HT29, and DLD1 cells	3, 10, 30, 40, 60, 120 μm	Incubation (48 h)	Dose-dependent and Time-dependent	[36]
TQ decreased cell viability TQ inhibited the activation of NF-κB by reducing the phosphorylation of its p65 subunit TQ down-regulated VEGF, c-Myc, and Bcl-2	HCT116 and COLO205 cells	20, 40, 60 μM	Incubation (24 h)	Dose-dependent	[35]
TQ reduced tumor counts large ACFs TQ downregulated Wnt, β-catenin, NF-κB, COX-2, TGF-β1, HSP-90, and VEGF and upregulated tumor suppressor genes like *DKK-1* and *CDKN1-A* TQ increased expression of smad4 and reduced iNOS	Male Wistar rats (200–250 g)	35 mg/kg/day	Oral gavage (3 times/week)	Dose-dependent	[28]
TQ increased the number of cells in sub-G1 phase TQ reduced hydrogen peroxide (H_2_O_2_) levels while increasing total GSH TQ induced the expression of BAX, Cytochrome C, and caspase-3 TQ reduced PTEN and inhibited PI3K/AKT/mTOR pathway	SW480 cells	75 μM	Incubation (12 h)	Dose-dependent	[26]
TQ reduced cell viability of cells TQ inhibited the expression of PI3K, Akt, p-GSK3β, β-catenin and COX-2 TQ inhibited the nuclear translocation of β-catenin TQ reduced cell migration	LoVo cells	2.5, 5, 7.5, 10, 20 μM	Incubation (24 h)	Dose-dependent	[13]
TQ reduced the total protein levels and phosphorylation of IKKα/β and NF-κB TQ decrease in protein levels of EMT markers Snail, Twist, MMP-2, and MMP-9 TQ reduced the cell migratory abilities of the cells	LoVo cells	2, 4, 6, 8 μM	Incubation (24 h)	Dose-dependent	[38]
TQ treatment reduced the viability of cells TQ treatment induced the cleavage of caspase-9, caspase-7, caspase-3, and PARP TQ treatment attenuated STAT3 activation and expression of its target gene products TQ inhibited STAT3 activation by blocking the phosphorylation of EGFR (Y1173), JAK2 and Src kinases	HCT-116 cells	10, 25, 50 μM	Incubation (24, 48, 72 h)	Dose-dependent and Time-dependent	[30]
TQ treatment caused early and dramatic increase in the amount of H2A.X protein TQ treatment upregulated the production of p53^+/+^ TQ treatment down regulated the production of CHEK1 TQ lowered CHEK1 expression and induced apoptosis in a p53-dependent manner	HCT116 cells Male NMRI mice (4–6 weeks)	60 μM 20 mg/kg	Incubation (24, 48, 72 h) Intraperitoneal injection (3 times/week)	Dose-dependent and Time-dependent	[15]
TQ elevated the total cell death index TQ reduced cytochrome c and cleaved caspase 3 TQ induced autophagic cell death via increasing MOMP and activating JNK and p38 TQ treatment increased the cellular expression of LC3	Lovo cells	2, 4, 6, 8 μM	Incubation (24 h)	Dose-dependent	[29]
TQ treatment reduces the gross tumour cell count TQ attenuated CRC initiation and enhanced the tumoricidal and chemopreventive efficacy of 5-FU Combined TQ and 5-FU treatment synergistically upregulated the expression of DKK-1, CDNK-1A, TGF-β1, and Smad4 Combined TQ and 5-FU therapy repressed the expression of β-catenin and iNOS	Adult Male Wistar rats (230 ± 20 g)	35 mg/kg/day	Orally by gastric gavage (3 times/week)	Dose-dependent	[27]
TQ increased the percentage of cells in the pre-G1 phase of the cell cycle TQ increased apoptosis, increasing caspase-3 and caspase-7 activity TQ activated the ERK and JNK pathways	HCT116, Caco-2, LoVo, and DLD-1 cells	20, 40, 60, 80, 100 μM	Incubation (24 and 48 h)	Dose-dependent and Time-dependent	[31]
TQ activated GSK-3β, inducing membranous localization of β-catenin and reduced nuclear c-myc expression TQ reduced the number of colonic polyps and their growth and induced apoptosis in polyps TQ reduces c-myc expression in the polyps TQ translocates β–catenin to the membrane in polyps	RKO cells Male and Female ApcMin mice (4–6 weeks old)	10, 15, 30, 60, 90 μM 375 mg/kg	Incubation (24 h) Fed with chow (12 weeks)	Dose-dependent	[12]
TQ decreased the concentration of GSH in its reduced form	Caco2 cells	389.60 µg/mL	Incubation (24 h)	Dose-dependent	[17]
TQ caused a significant decrease in cell viability TQ induced an increase in LDH release TQ increased the percentage of apoptotic cells	HCT116 and HT-29 cells	12.5, 25, 50, 100 μg/mL	Incubation (24 and 48 h)	Dose-dependent	[20]
TQ reduced cell viability TQ decreased cell proliferation rate TQ treatment reduces glycolytic metabolism/glucose fermentation rate (Warburg effect) TQ inhibited HK2 expression and PI3K-AKT activation	HCT116 and SW480 cells	20.53 and 21.71 µM	Incubation (4 days)	Dose-dependent and Time-dependent	[18]
TQ led to PARP cleavage	HCT116 and HT-29 cells	1, 2.5, 5 μM	Incubation (24 h)	Dose-dependent	[22]
TQ reduces cell viability TQ significantly decreased the sphere formation ability TQ decreased expression CD44, EpCAM, and Ki67 TQ inhibited cell migration ability by upregulating E-cadherin and downregulating mesenchymal markers TQ caused an upregulation of p53, p21, γ-H2AX TQ inhibited tumor growth	HCT116 cells Male NOD-SCID and NOG mice (6–8 weeks old)	1, 3, 5, 40, 60, 100 μM 20 mg/kg	Incubation (24, 48, 72 h) Intraperitoneal injection (3 times/week for 21 days)	Dose-dependent and Time-dependent Time-dependent	[16]
TQ significantly decreased cell viability	HCT-116 cells	60 and 68 μM	Incubation (24 h)	Dose-dependent	[14]
TQ reduced cell growth and improved replication fidelity TQ reduced small intestinal tumor incidence TQ reduced the number of small and medium-sized intestinal tumors TQ decreased mutation rates	HCT116 cells Male and Female Msh2loxP/loxP Villin-Cre mice (4–6 weeks)	1.25 and 2.50 μM 37.5 and 375 mg/kg	Incubation (7 days) Fed through chow (43 weeks)	Dose-dependent Dose-dependent	[37]
TQ significantly increased cell mortality rate TQ treatment decreased cell proliferation TQ treatment induced morphological changes in cells by inducing p53 overexpression and caspase 3/Mcl-1 downregulation	HCT116 cells	20 and 60 μM	Incubation (20 h)	Dose-dependent	[19]
TQ inhibited cell proliferation TQ induced significant alterations in cellular morphology TQ induced cell cycle arrest at G1/S phase TQ upregulated p53 mRNA expression and decreased Bcl-2	HCT116 cells	10–100 μM	Incubation (12, 24, 48 h)	Dose-dependent and Time-dependent	[25]
TQ decreased cellular viability TQ reduced the invasive abilities of the cells TQ downregulated the expression of ATG-12, ATG-7, and LC3-II TQ downregulated the expression of Bcl-2 and Bcl-X and upregulated Bax	HT-29 cells	19, 18, 39, 75, 150 µM	Incubation (6, 24, 48 h)	Dose-dependent and Time-dependent	[24]
TQ reduced cell viability	HT-29 cells	3, 5, 8 μg/m	Incubation (24 and 72 h)	Dose-dependent and Time-dependent	[21]
TQ treatment reduced the number and sizes of aberrant crypt foci (ACF) TQ reduced cell invasion potential of cells. TQ displayed increased apoptosis, loss of intercellular adhesion, and sponge-like growth pattern TQ treatment significantly reduced tumor multiplicity and size	C26 cells Female BALB/c mice (20–24 g, 9 weeks)	20, 40, 60 μM 5, 10, 20, 30 mg/kg	Incubation (24 h) Intraperitoneal injection (daily for 20 days)	Dose-dependent Dose-dependent	[23]
TQ reduced tumor incidence, multiplicity, and size TQ attenuated oxidative stress by reducing ROS and restoring antioxidant enzyme levels of GPx, CAT, SOD, and GSH	Adult Male Wistar rats	5 mg/kg	Intraperitoneal injection (once every 10 or 20 weeks)	Time-dependent	[32]
TQ increased Bax and decreased Bcl-2, inducing apoptosis TQ inhibited expression and activity of NF-κB and STAT3	HCT116 cells	30 μM	Incubation (24 h)	-	[40]

## Data Availability

The datasets used and/or analyzed during the current study are available from the corresponding author on reasonable request.
